# Supplementary material for: Childbirth Care among SARS-CoV-2 Positive Women in Italy
Source: Int J Environ Res Public Health. 2021 Apr 16;18(8):4244. doi: 10.3390/ijerph18084244 (PMC8074190; doi:10.3390/ijerph18084244)
Supplement: Supplementary file 1 [file ijerph-18-04244-s001.zip › ijerph-1175161-supplementary.pdf]

ItOSS National network of maternity units

Piedmont Region

Elena Amoruso *Ospedale Sant'Andrea Vercelli;* Alberto Arnulfo, Enrico Finale *Stabilimento Ospedaliero Castelli Verbania;* Rossella Attini, Marisa Biasio, Luca Marozio, Clara Monzeglio OIRM *Sant'Anna - AOU Città della Salute e della Scienza di Torino;* Maria Bertolino, Andrea Guala *Ospedale San Biagio Domodossola;* Silvia Bonassisa, Alberto De Pedrini *Ospedale Maggiore della Carità Novara;* Mario Canesi, Sara Cantoira *Ospedale Maria Vittoria Torino;* Paola Capelli *Istituto SS. Trinità Borgomanero;* Ilaria Careri, *Ospedale Martini Torino;* Luigi Carratta *Ospedale S. Spirito Casale Monferrato;* Ilaria Costaggini *Ospedale degli Infermi Rivoli;* Tania Cunzolo *Presidio Osp. Cardinal G. MASSAIA Asti;* Enza De Fabiani, Andrea Villasco *Azienda Ospedaliera Ordine Mauriziano Torino;* Cinzia Diano *Ospedale Maggiore Chieri;* Fiorenza Droghini, Paola Rota *Ospedale Santa Croce Moncalieri;* Daniela Kozel, Vittorio Aguggia *Ospedale Civile SS. Antonio e Biagio Alessandria;* Francesca Maraucci *Ospedale degli infermi Biella;* Gisella Martinotti *Ospedale SS. Pietro e Paolo Borgosesia;* Maria Milano, Antonia Novelli *Ospedale Civile Mondovì;* Giovanna Oggè *Ospedale maggiore SS. Annunziata Savigliano;* Simona Pelissetto *Ospedale Civile di Ivrea;* Pasqualina Russo *Presidio Osp. riunito Ciriè;* Manuela Scatà *Ospedale Michele e Pietro Ferrero di Verduno;* Federico Tuo, Valentina Casagrande *Ospedale San Giacomo Novi Ligure/Tortona;* Concetta Vardè *Ospedale Agnelli Pinerolo;* Elena Vasario *Azienda Ospedaliera S. Croce e Carle Cuneo*; Daniela Ventrella *Ospedale Civico Chivasso*

Valle D'Aosta Region

Livio Leo *Umberto Parini Aosta*

Liguria Region

Silvia Andrietti *ASL1 Imperiese*; Federica Baldi *Ospedale San Paolo Savona*; Angelo Cagnacci, Federica Laraud *IRCCS AOU San Martino*; Franco Camandona, Domenico Grimaldi *Ospedale Galliera di Genova*; Maria Franca Corona, Massimiliano Leoni *Ospedale Civile Sant'Andrea La Spezia*; Paolo Massirio, Luca Ramenghi *IRCCS Giannina Gaslini*

Lombardy Region

Debora Balestrieri *Ospedale di Cittiglio;* Federica Baltaro *Ospedale Niguarda di Milano;* Pietro Barbacini, Elisabetta Venegoni *Ospedale di Magenta;* Michele Barbato *Ospedale di Melegnano;* Lorena Barbetti *Ospedale di Esine;* Paolo Beretta *Ospedale di Como;* Bruno Bersellini *Ospedale di Sondrio;* Stefano Bianchi *Ospedale San Giuseppe di Milano;* Antonia Botrugno *Ospedale di Casalmaggiore;* Donatella Bresciani *Ospedale di Desenzano;* Alessandro Bulfoni *Pio X Humanitas di Milano;* Carlo Bulgheroni *Ospedale di Gallarate;* Orlando Caruso, Elena Pinton *Ospedale di Chiari;* Massimo Ciammella *Ospedale di Seriate;* Elena Crestani, Giulia Pellizzari *Ospedale di Pieve di Coriano;* Antonella Cromi *Ospedale di Varese;* Serena Dalzero, Nikita Alfieri *Ospedale San Paolo di Milano;* Rosa Di Lauro, Carla Foppoli *Ospedale di Sondalo;* Patrizia D'Oria, *Ospedale di Alzano;* Santina Ermito *Ospedale di Piario;* Massimo Ferdico *Ospedale di Vimercate;* Maria Fogliani, Guido Stevanazzi *Ospedale di Legnano-Cuggiono;* Roberto Fogliani *Ospedale di Sesto San Giovanni;* Ambrogio Frigerio *Ospedale di Rho;* Eleonora Fumagalli *Ospedale Macedonio Melloni ASST FBF-Sacco di Milano;* Roberto Garbelli *Brescia Istituto Clinico S. Anna;* Daniela Gatti *Ospedale di Manerbio;* Giampaolo Grisolia, Serena Varalta *Ospedale di Mantova;* Paolo Guarnerio *Ospedale San Carlo di Milano;* Enrico Iurlaro, Marta Tondo *IRCCS Cà Granda Ospedale Maggiore Policlinico-Mangiagalli Milano;* Stefano Landi *Ospedale di Gravedona;* Mario Leonardi *Ospedale di Iseo;* Stefania Livio, Chiara Tasca *Ospedale Buzzi ASST FBF-Sacco di Milano;* Anna Locatelli *Ospedale di Carate;* Giuseppe Losa *Ospedale di Melzo;* Massimo Lovotti *Como Valduce;* Anna Minelli *Ospedale di Gavardo;* Luisa Muggiasca *Ospedale di Garbagnate;* Giuseppe Nucera *Ospedale di Busto Arsizio;* Alessandra Ornati *Ospedale di*

Vigevano; Luisa Patanè *ASST Papa Giovanni XXIII Bergamo;* Antonio Pellegrino *Ospedale di Lecco;* Francesca Perotti, Arsenio Spinillo *Fondazione IRCCS Policlinico San Matteo di Pavia;* Armando Pintucci *Ospedale di Desio;* Ezio Pozzi *Ospedale di Broni Stradella- Ospedale di Voghera,* Federico Prefumo *Spedali Civili di Brescia;* Anna Catalano *Brescia Fondazione Poliambulanza;* Aldo Riccardi *Ospedale di Cremona;* Alessia Chiesa *Ospedale di Ponte San Pietro;* Tazio Sacconi *Ospedale di Asola;* Valeria Savasi, Silvia Corti *Ospedale Sacco di Milano;* Ubaldo Seghezzi *Ospedale di Saronno;* Vincenzo Siliprandi *Ospedale di Crema;* Marco Soligo, Beatrice Negri *Ospedale di Lodi;* Paolo Valsecchi *Ospedale San Raffaele;* Laura Vassena *Ospedale di Merate;* Federica Brunetti, Patrizia Vergani *Fondazione MBBM Ospedale San Gerardo Monza;* Antonella Villa *Ospedale di Treviglio;* Matteo Zanfrà *Ospedale di Tradate;* Alberto Zanini *Ospedale di Erba*

Autonomous province of Bozen

Martin Steinkasser, Micaela Veneziano *Ospedale Centrale di Bolzano*

Autonomous province of Trento

Pietro Dal Rì, Fabrizio Taddei *UO Rovereto*; Roberto Luzietti *UO Cles*; Saverio Tateo *UO Trento*; Fabrizia Tenaglia *UO Cavalese*

Veneto Region

Giuseppe Angeloni *Ospedale di Piove di Sacco;* Antonio Azzena *Ospedale di Vittorio Veneto;* Gianluca Babbo *Ospedale di Portogruaro*; Roberto Baccichet, Cristina Napolitano *Ospedale di Oderzo;* Valentino Bergamini *Ospedale Borgo Trento;* Luca Bergamini *Ospedale di Chioggia;* Enrico Busato, Monica Zannol *Ospedale di Treviso;* Pietro Catapano, Marco Gentile *Ospedale Mater Salutis – Legnago;* Marcello Ceccaroni *Ospedale Sacro Cuore don Calabria Negrar;* Gianluca Cerri *Ospedale SS. Giovanni e Paolo – Venezia;* Andrea Cocco *Ospedale di Asiago;* Carlo Dorizzi *Ospedale di Schiavonia;* Laura Favretti *Ospedale S. Maria del Prato – Feltre;* Riccardo Federle, Antonino Lo Re *Ospedale P. Pederzoli - Casa di cura Privata Spa;* Massimo Franchi, Marina Sangaletti *Azienda Ospedaliera di Verona;* Franco Garbin *Ospedale di Dolo;* Maria Teresa Gervasi, Daniela Truscia *Azienda Ospedaliera di Padova;* Dimosthenis Kaloudis *Ospedale di San Bonifacio;* Domenico Lagamba *Ospedale di Castelfranco Veneto;* Giovanni Martini *Ospedale di Valdagno;* Carlo Maurizio *Ospedale di Mirano;* Yoram J. Meir *Ospedale di Bassano del Grappa;* Alessia Pozzato *Ospedale di Adria* - *Ospedale di Rovigo;* Marcello Rigano *Ospedale di Camposampiero;* Cesare Romagnolo *Ospedale all'Angelo di Mestre;* Roberto Rulli *Ospedale di Cittadella;* Giuseppe Sacco *Ospedale di San Donà di Piave;* Maria Grazia Salmeri *Ospedale di Montebelluna;* Marcello Scollo *Ospedale di Santorso;* Francesco Sinatra *Ospedale di Conegliano;* Gianluca Straface *Casa di cura Abano;* Fabio Gianpaolo Tandurella *Ospedale di Pieve di Cadore* e *Ospedale San Martino – Belluno;* Marco Torrazzina *Ospedale di Bussolengo* - *Ospedale di Villafranca;* Paolo Lucio Tumaini *Ospedale di Arzignano;* Giuliano Zanni *Ospedale di Vicenza*

Friuli-Venezia Giulia Region

Emanuele Ancona Ospedale *S. Giorgio di Pordenone;* Michela De Agostini *Ospedale di Palmanova;* Gianpaolo Maso, Alice Sorz *IRCSS Burlo Garofolo Trieste;* Edlira Muharremi *S. M. degli Angeli Ospedale di Pordenone;* Alessandra Nicoletti *Ospedale S.Daniele di Tolmezzo;* Roberta Pinzano *Ospedale S.Maria dei Battuti-San Vito al Tagliamento;* Alessia Sala O*spedale Santa Maria della Misericordia-Udine;* Lucia Zanazzo, *Ospedale di Monfalcone*

Emilia-Romagna Region

Lorenzo Aguzzoli, Alice Ferretti *Ospedale S.M. Nuova Reggio Emilia;* Patrizio Antonazzo, Lucrezia Pignatti *Ospedale Bufalini Cesena;* Angela Bandini, Isabella Strada *Ospedale G.B .Morgagni -L. Pierantoni Forlì;* Chiara Belosi *Ospedale degli Infermi Faenza;* Renza Bonini, Maria Cristina Ottoboni *Ospedale Guglielmo Da Saliceto Piacenza;* Fabrizio Corazza, Paola Pennacchioni *Ospedale Ss. Annunziata Cento;* Fabio Facchinetti,

Giliana Ternelli *Azienda Ospedaliero-Universitaria Modena*; Alessandro Ferrari, Cristina Pizzi, *Ospedale S.M. Bianca Mirandola;* Tiziana Frusca, Stefania Fieni *Azienda Ospedaliero-Universitaria Parma;* Maria Cristina Galassi, Federica Richieri, *Nuovo Ospedale Civile Di Sassuolo S.P.A.;* Francesco Giambelli, Carlotta Matteucci *Ospedale S.M. Delle Croci Ravenna;* Pantaleo Greco, Danila Morano *Azienda Ospedaliero-Universitaria Ferrara;* Marinella Lenzi, Ilaria Cataneo, *Ospedale Maggiore Bologna;* Gialuigi Pilu, Marisa Bisulli, *Azienda Ospedaliero-Universitaria Bologna;* Maria Cristina Selleri *Ospedale di Bentivoglio;* Federico Spelzini, Elena De Ambrosi, *Ospedale Infermi Rimini;* Paolo Venturini, Francesca Tassinati *Ospedale B. Ramazzini Carpi;* Stefano Zucchini, Barbara Paccaloni, *Ospedale S.M. della Scaletta Imola*

Tuscany Region

Andrea Antonelli, Carlotta Boni *Ospedale Civile Cecina;* Maria Paola Belluomini, *S. Francesco Barga - PO Valle del Serchio* e *Generale Provinciale Lucca - PO San Luca,* Rosalia Bonura, *S. Maria della Gruccia - Ospedale del Valdarno,* Stefano Braccini, *SS. Cosimo e Damiano Pescia - Osp della Valdinievole,* Giacomo Bruscoli e Pasquale Mario Florio, *Nuovo Ospedale San Jacopo di Pistoia,* Giovanna Casilla, *SS. Giacomo e Cristoforo Massa - PO Zona Apuana,* Anna Franca Cavaliere, *Ospedale Santo Stefano Prato,* Marco Cencini, *Ospedali Riuniti della Val di Chiana,* Venere Coppola e Laura Migliavacca, *Ospedale Misericordia Grosseto,* Barbara De Santi, *PO Felice Lotti Pontedera,* Paola Del Carlo, *Ospedale S.Giovanni Di Dio Torregalli,* Carlo Dettori, *Nuovo Ospedale di Borgo S.Lorenzo,* Mariarosaria Di Tommaso e Serena Simeone, *Careggi - CTO Firenze - AOU,* Giuseppe Eremita, *Civile Elbano Portoferraio,* Sara Failli, *Ospedale Area Aretina Nord Arezzo,* Paolo Gacci, *S.M. Annunziata Bagno a Ripoli - Osp Fiorentino Sud Est,* Alessandra Meucci, *Le Scotte Siena - Azienda ospedaliera universitaria,* Filippo Ninni, *Riuniti Livorno,* Barbara Quirici, *Ospedale Unico Versilia,* Alessia Sacchi, *Ospedale dell'Alta Val d'Elsa Poggibonsi,* Cristina Salvestroni, *Ospedale S. Giuseppe Empoli,* Sara Zullino, *Ospedali Pisani Pisa - Az universitaria*

Umbria Region

Nazzareno Cruciani, Fabrizio Damiani *Ospedale San Giovanni Battista Foligno*; Leonardo Borrello *Azienda Ospedaliera Santa Maria di Terni*; Gian Carlo Di Renzo, Giorgio Epicoco *Azienda Ospedaliera Santa Maria della Misericordia di Perugia*; Ugo Indraccolo, Donatello Torrioli, *Ospedale di Città di Castello*

Marche Region

Andrea Ciavattini, Sara D'Eusanio *AOU - Ospedali Riuniti di Ancona*; Filiberto Di Prospero *Ospedale di Civitanova Marche*; Rebecca Micheletti, Claudio Cicoli *Azienda Ospedaliera Ospedali Riuniti Marche Nord*

Lazio Region

Francesco Antonino Battaglia, Immacolata Marcucci *PO Santa Maria Goretti Latina;* Leonardo Boccuzzi, Patrizia Ruocco *Ospedale De Santis di Genzano*; Marco Bonito *Ospedale San Pietro Fatebenefratelli Roma;* Maria Clara D'Alessio *San Filippo Neri Roma*; Carlo De Angelis *Casa di Cura Fabia Mater Roma*; Donatella Dell'Anna *Ospedale S. Eugenio*; Daniele Di Mascio, Paola Pecilli *Umberto I - Policlinico di Roma;* Sergio Ferrazzani *Policlinico Universitario Fondazione Agostino Gemelli - Roma;* Gregorio Marco Galati *Ospedale Madre Giuseppina Vannini Istituto delle Figlie di S. Camillo Roma*; Maria Grazia Frigo *Fatebenefratelli San Giovanni Calibita - Isola Tiberina;* Paolo Gastaldi *Ospedale Santo Spirito Roma*; Rita Gentile *Presidio Ospedaliero Giovan Battista Grassi Ostia;* Giovanni Grossi *Ospedale Sandro Pertini Roma*; Giorgio Nicolanti, Patrizio Raggi *Ospedale Belcolle Viterbo;* Flavia Pierucci *Azienda Ospedaliera San Camillo Forlanini Roma*; Giancarlo Paradisi, Maria Rita Pecci *Ospedale Fabrizio Spaziani Frosinone*; Giovanni Testa *Casa di cura Città di Aprilia*; Barbara Vasapollo *Policlinico Casilino Roma;* Barbara Villaccio *Ospedale San Pietro Fatebenefratelli Roma*

Abruzzo Region

Fabio Benucci *Sant'Omero;* Paola Caputo *Sulmona;* Sandra Di Fabio, Maurizio Guido *L'Aquila;* Antonio Di Francesco *Lanciano;* Francesca di Sebastiano, Diego Gazzolo, Marco Liberati *Chieti;* Anna Marcozzi *Teramo;* Francesco Matrullo *Vasto;* Maurizio Rosati, Gabriella Scorpio *Pescara;* Giuseppe Ruggeri *Avezzano*; Alessandro Santarelli *Ospedale di Sant'Omero*

Molise Region

Daniela Simeone *Ospedale di Campobasso*

Campania Region

Annalisa Agangi *Ospedale Evangelico Villa Betania*; Salvatore Ercolano *P.O. "S. Leonardo" di Castellammare di Stabia*, Luigi Cobellis, Annunziata Mastrogiacomo *Ospedale di Caserta*; Maria Vittoria Locci *AOU Federico II Napoli*

Puglia Region

Luca Loiudice *Bari - Presidio Mater Dei;* Antonio Belpiede *Barletta 'Mons. Dimiccoli';* Mariano Cantatore *L. Bonomo Andria;* Ettore Cicinelli, Antonella Vimercati *Bari - Policlinico Ginecologia;* Aldo D'Aloia, Sabina Di Biase, Antonio Lacerenza *AOU "OO RR Foggia";* Alessandro Dalfiero *Cerignola;* Gerardo D'Ambrogio *Galatina "Santa Caterina Novella";* Nicola Del Gaudio *Castellaneta;* Paolo Demarzo *San Severo Teresa Masselli Mascia;* Giovanni Di Vagno *Bari - San Paolo;* Giuseppe Laurelli *Casa Sollievo Dalla Sofferenza - S. Giovanni Rotondo;* Roberto Lupo *Gallipoli;* Nicola Macario *Altamura;* Antonio Malvasi, *Bari - Casa Di Cura Santa Maria;* Guido Maurizio, Elisabetta Monteduro, *Acquaviva "Miulli";* Andrea Morciano *Cardinale G. Panico Di Tricase;* Lucio Nichilo *Umberto I Corato;* Anna Maria Nimis *Francavilla Fontana;* Antonio Perrone *Lecce Vito Fazzi;* Elena Rosa Potì *Brindisi "Perrino";* Sabino Santamato *Monopoli Putignano;* Emilio Stola *Taranto;* Antonio Tau *Scorrano;* Mario Vicino *Bari - Di Venere;* Martino Vinci *Martina Franca*

Basilicata Region

Giampiero Adornato *Policoro*; Francesco Bernasconi *Melfi*; Alfonso Chiacchio *Lagonegro*; Sergio Schettini, Rocco Paradiso *Azienda Ospedaliera Regionale San Carlo – Potenza*; Giuseppe Trojano *Matera*

Calabria Region

Carmelina Ermio *Ospedale Jazzolino - Vibo Valentia*; Michele Morelli, Rossella Marzullo *Ospedale Annunziata – AO Cosenza;* Stefano Palomba *Ospedali Riuniti di Reggio Calabria;* Morena Rocca *Azienda Ospedaliera "Pugliese Ciaccio" di Catanzaro*

Sicily Region

Vincenzo Aidala, *Castiglione Prestianni-Bronte;* Luigi Alio, Giuseppina Orlando *ARNAS Civico di Cristina Benfratelli-Palermo;* Maria Grazia Arena, Santo Recupero *S. Marco (ex V. Emanuele S. Bambino)-Catania* e *Osp. Generale-Lentini;* Salvatore Bevilacqua, Fabrizio Quartararo *Casa di cura Candela SPA-Palermo;* Rocco Billone *Civico Partinico* e *Dei Bianchi-Corleone;* Giuseppe Bonanno, *Maria Paternò Arezzo-Ragusa;* Antonio Bucolo, *Umberto I-Siracusa;* Claudio Campione, *Casa di cura prof. Falcidia-Catania;* Giuseppe Canzone, *S. Cimino-Termini Imerese;* Angelo Caradonna, *V. Emanuele II-Castelvetrano;* Sebastiano Caudullo e Cosimo Raffone, *AO Papardo-Messina;* Giovanni Cavallo, *PO Maggiore-Modica;* Antonio Cianci, Michele Fichera *V. Emanuele Rodolico-Catania;* Salvatore Corsello, Sergio Di Salvo *Casa di cura Villa Serena-Palermo;* Gaspare Cucinella, Maria Elena Mugavero *Villa Sofia - Cervello-Palermo;* Rosario D'Anna, *AOU G. Martino-Taormina,* Maria Rosa D'anna, *Buccheri La Ferla-Palermo;* Maria Di Costa, *Basilotta-Catania;* Giuseppe Ettore, *ARNAS Garibaldi Nesima-Catania;* Giovanni Falzone, Marta Fauzia *Umberto I-Nicosia;* Roberto

Fazio, *G. Fogliani-Milazzo* e *Lipari;* Matteo Giardina *Ospedale Paolo Borsellino di Marsala*; Michele Gulizzi e Francesco La Mantia, *G. F. Ingrassia-Palermo;* Laura Giambanco, *S. Antonio Abate-Erice* e *B. Nagar-Pantelleria;* Salvatore Incandela, *S. Giovanni di Dio -Agrigento* e *Giovanni Paolo II-Sciacca;* Lilli Maria Klein *S. Vincenzo-Enna* e *Barone Romeo-Patti;* Michele La Greca, Venera Mille, *M. SS. Addolorata-Biancavilla;* Luigi Li Calsi *S. Giacomo d'Altopasso-Licata;* Emilio Lo Meo, Paolo Scrollo *Cannizzaro-Catania;* Vincenzo Miceli, *S. Raffaele Giglio-Cefalù;* Maria Pia Militello, *S. Marta e S. Venera-Acireale;* Alfio Mirenna, *Istituto clinico Vidimura (ex Casa di cura Gretter e Lucina)-Catania;* Pietro Musso, *Abele Ajello-Mazara del Vallo;* Michele Palmieri, *V. Emanuele-Gela;* Concetta Remigia Pettinato, Angelo Tarascio *Gravina - Caltagirone-Caltagirone;* Vincenzo Scattarreggia *Barone Lombardo-Canicattì;* Antonio Schifano *R. Guzzardi-Vittoria;* Calogero Selvaggio *S. Elia-Caltanissetta;* Luigi Triolo *Casa di cure Triolo Zancla SPA-Palermo;* Renato Venezia *P. Giaccone-Palermo*

Sardinia Region

Speranza Piredda *Civile Alghero;* Giangavino Peppi *Giovanni Paolo II - Olbia;* Giovanna Pittorra *S. Fracesco - Nuoro;* Gianfranco Depau *Nostra Signora della Mercede - Lanusei;* Gianfranco Puggioni *S. Martino - Oristano;* Loredana Pagliara *Nostra Signora di Bonaria - San Gavino;* Giulietta Ibba *CTO - Iglesias;* Caterina Tronci, *SS Trinità - Cagliari;* Giampiero Capobianco, *AOU Sassari;* Alessandra Meloni *Duilio Casula Monserrato AOU - Cagliari;* Francesca Palla *S. Michele AO Brotzu – Cagliari*
